# Interface Characteristics and Mechanical Properties of Ultrasonic-Assisted Friction Stir Lap Welded 7075-T6 Aluminium Alloy

**DOI:** 10.3390/ma13235335

**Published:** 2020-11-25

**Authors:** Changshu He, Zhiqiang Zhang, Ying Li, Jingxun Wei, Menggang Zhai, Su Zhao, Xiang Zhao

**Affiliations:** 1 School of Materials Science and Engineering, Northeastern University, Shenyang 110819, China; lnkdzzq@126.com (Z.Z.); liying3273@163.com (Y.L.); 18978226891@163.com (J.W.); zhaox@mail.neu.edu.cn (X.Z.); 2 Key Laboratory for Anisotropy and Texture of Materials, Northeastern University, Shenyang 110819, China; 3 Ningbo Institute of Materials Technology & Engineering, Chinese Academy of Sciences, Ningbo 315201, China; zhaimgmail@nimte.ac.cn (M.Z.); zhaosu@nimte.ac.cn (S.Z.)

**Keywords:** ultrasonic-assisted friction stir lap welding, material flow, microstructure, shear fracture strength, fracture mode

## Abstract

In this work, friction stir lap welding (FSLW) and ultrasonic-assisted friction stir lap welding (UAFSLW) was applied to 6-mm-thick 7075-T6 alloy sheets using three welding tools with the same process parameters. The joint formation, microstructural characteristics, and mechanical properties of the resulting lap joints were then investigated. The results showed that ultrasonic vibration significantly promoted the flow of metal at the interface, enlarged the size of the stirred zone (SZ), and reduced the angle between the hook defect and the interface. During lap shear testing, the FSLW and UAFSLW joints fractured in a similar manner. The fracture modes included tensile fracture, shear fracture, and a mixture of both. Cold lap and hook defects may have served as crack-initiation zones within the joint. Under configuration A (i.e., upper sheet on the retreating side (RS)), all joints failed in the shear-fracture mode. The effective lap width (ELW) of the joint welded using tool T2 was the greatest. This resulted in a higher shear fracture strength. The maximum shear fracture strength of the UAFSLW joint was 663.1 N/mm. Under configuration B (i.e., upper sheet on the advancing side (AS)), the shear fracture strength was greatly affected by the fracture mode. The highest shear fracture strength of the UAFSLW joint, 543.7 N/mm, was welded by tool T3. Thus, under otherwise identical conditions, UAFSLW joints can withstand a greater fracture shear strength than FSLW joints, as ultrasonic vibration helps to mix the material at the interface, thus, enlarging the SZ and diminishing the cold lap defects.

## 1. Introduction

In the 1990s, The Welding Institute (TWI, United Kingdom) invented friction stir welding, which is characterised by less distortion, fewer defects, and lower production costs and is suitable for joining light alloys [1,2,3]. FSW includes friction stir butt welding and friction stir lap welding (FSLW) [4,5] in which the latter is the focus of the present work. However, the different positions of the faying surface and the bending deformation at the lap interface of FSLW joints may cause interface defects, including hook defects and cold lap defects. The presence of these defects generally reduces the effective sheet thickness and effective lap width (ELW), thereby degrading the tensile shear properties of FSLW joints [6,7,8].

The interface defect morphology is significantly affected by the rotational speed, welding speed, and pin geometry [9,10,11,12]. Chen et al. [13] investigated how, as a function of rotational speed, pin length affects the mechanical properties of FSLW joints of 1.4-mm-thick 2A97 aluminium alloy and found that the joint failure load decreased with increasing pin length and rotational speed. Wang et al. [14] found that, as the pin length increases, the hook defect on the advancing side (AS) evolves following an “M” trend, i.e., as the pin length increased from 2.0 to 3.6 mm, the hook size increased, decreased, increased, and then decreased again. The impact of the pin length on the joint properties depended on the heat input. Song et al. investigated how the process conditions (i.e., welding speed and joint combination) affected defect features and the mechanical properties of joints [15]. Their results showed that, in both combinations, hook defects protruded upward into the stirred zone (SZ) at lower welding speeds, and the lap shear strength generally increased with welding speed. Li et al. [16] produced FSLW joints of 2024-T4 aluminium alloy by an external stationary shoulder, which was beneficial to joint formation and produced joints with a thinner effective sheet and a wider effective lap, thereby increasing the shear-failure strength. These studies mainly focused on improving the flow behaviour of the material at the interface by optimising the welding parameters and tools to reduce the size of interface defects and improve the mechanical properties of FSLW joints.

Ultrasonic vibration causes stress superposition and acoustic softening, which significantly reduces the yield strength of the material during tension or compression [17,18]. In recent years, ultrasonic vibration has been combined with friction stir welding in the form of ultrasonic-assisted friction stir welding. Applying ultrasonic vibration during the welding process offers the advantages of expanding the welding-process window, reducing defects and improving the mechanical properties of the joint [19,20,21,22,23,24,25,26]. Currently, researchers have also introduced ultrasonic vibration into the FSLW process. For example, Ji et al. [27] developed Zn-added ultrasonic-assisted friction stir lap welding (UAFSLW) of Al/Mg alloys in which the ultrasonic probe was attached to the bottom surface of the lower plate. Their results showed that finer and better-distributed Mg-Zn intermetallic compounds in the joint produced by Zn-added UAFSLW increased the maximum tensile shear load of the joint over that of a conventional joint by 53%. Kumar et al. [28] applied horizontal ultrasonic vibration to weld Al-Mg FSLW joints and found that this eliminated defects and reduced the torque and load during the welding process. The failure load and elongation improved by 38% and 39%, respectively. Gao et al. [29] applied ultrasonic vibration ahead of the tool in FSLW of the 6061-T6 alloy. Ultrasonic vibration improved material flow, reduced interfacial defects, widened the ELW, shortened hooks, and increased joint strength.

The axial application of ultrasonic-assisted friction stir welding ensures the most efficient use of ultrasonic energy. In this technique, the ultrasonic amplitude rod is connected with the tool, and the weld is ultrasonically vibrated during welding. However, given that few reports exist on axial ultrasonic-assisted FSLW, investigations are needed to determine how axial ultrasonic vibration affects joint formation and the mechanical properties of joints. Thus, the present study uses three tools for conventional FSLW and axial ultrasonic-assisted FSLW of 6-mm-thick 7075-T6 aluminium alloys. We discussed in detail the metal flow at the interface and the microstructure evolution and fracture behaviour under different loading configurations.

## 2. Materials and Methods

**Figure 1:**
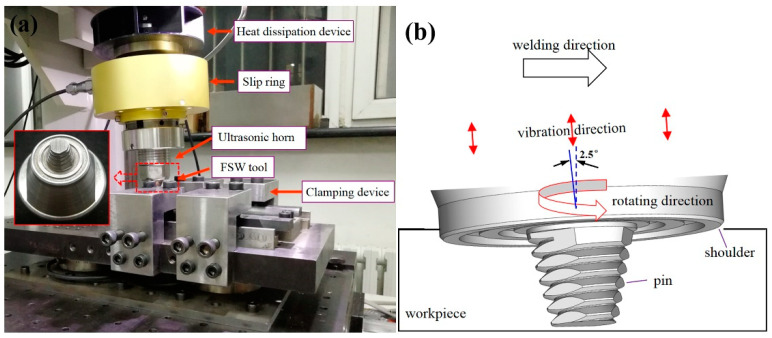
(**a**) Experimental apparatus and (**b**) schematic apparatus for axial ultrasonic-vibration-assisted friction stir lap welding (FSLW).

**Figure 2:**
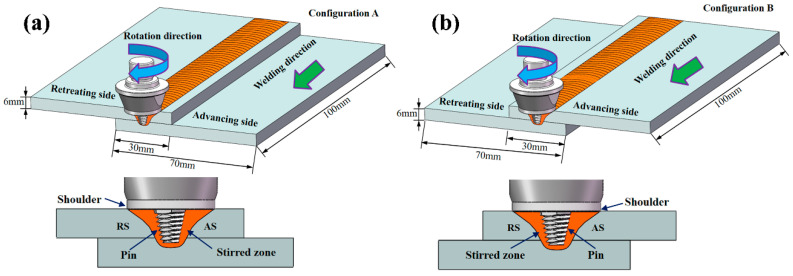
Schematic of lap joints in (**a**) configuration A and (**b**) configuration B.

**Figure 3:**
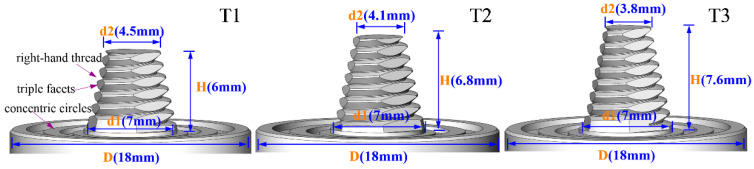
Profiles and main dimensions of tools used in this investigation.

This study used 6-mm-thick 7075-T6 aluminium alloy sheets as base material. The chemical composition and mechanical properties of the base material are summarised in Table 1. Two sheets with dimensions of 100 mm × 70 mm were joined at an overlap joint with an overlap width of 30 mm. The welding direction was parallel to the rolling direction of the base material. Before welding, the overlapping surfaces of the sheets were cleaned with sandpapers to remove oxidation and organic residue. The sheets were then fixed on a worktable, and lap joints were made by using a welding machine. Figure 1a shows the experimental setup for UAFSLW. For this experiment, an ultrasonic transducer was integrated with the rotating tool holder to vibrate the tool in the axial direction while the tool rotates (Figure 1b), transferring the ultrasonic energy into the weld. The frequency of the ultrasonic vibration was 20 kHz, and the maximum vibration amplitude was 10 µm. The difference in the metal flow behaviour of the AS and retreating side (RS) during FSLW directly affects the characteristics of the cold lap and hook defects. Therefore, two configurations were investigated, as shown in Figure 2. In configuration A, the upper sheet was on the RS. In configuration B, the upper sheet was on the AS. During the tensile process, the two configurations lead to a difference in the maximum stress of the AS and RS, which affects the fracture behaviour of the joint. FSLW and UAFSLW processes were performed under a fixed combination of process parameters. The tilting angle of the tool and the shoulder plunge depth were set at 2.5° and 0.2 mm, respectively. The rotation and welding speeds were constant at 1000 rpm and 70 mm/min, respectively. The shoulder diameter and geometry were the same for the three tools (T1, T2, and T3). See Figure 3 for the detailed geometry of the tools. To better observe the interface-metal flow characteristics of FSLW and UAFSLW joints welded by different tools, a pure-aluminium foil with a thickness of 0.1 mm was placed as a marker material between the upper and lower sheets. 

**Figure 4:**
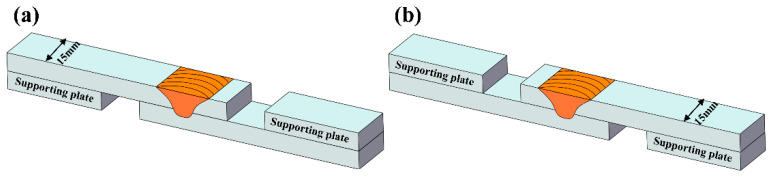
Schematic of tensile shear specimens: (**a**) configuration A and (**b**) configuration B.

After welding, transverse specimens for microstructural imaging were cut perpendicularly to the rolling direction and then mechanically ground and etched with Keller’s reagent (2 mL HF + 3 mL HCl + 5 mL HNO_3_ + 190 mL H_2_O). Metallographic observations were done by optical microscopy (Olympus-GX71, Olympus, Tokyo, Japan) and the morphology and distribution of the aluminium foil were investigated. Electron backscatter diffraction (EBSD) analysis was performed via a TESCAN MAIA3 scanning electron microscope (TESCAN, Brno, Czech Republic) using an acceleration voltage of 20 kV, and a step size of 0.35 μm. The specimens were prepared by electrolytic polishing in a solution of 20% H_2_SO_4_ and 80% CH_3_OH at 20 V for 20 s. The EBSD data were processed using HKL Channel 5 software. The grain boundaries were composed of low-angle grain boundaries (from 5° to 15°) and high-angle grain boundaries (over 15°), denoted by green and black lines, respectively. To evaluate the mechanical properties of the lap joints, lap shear tests were performed in accordance with the Japanese Industrial Standard (JIS) Z 3136-1999 using a Shimadzu AG-X Plus universal testing machine (Tokyo, Japan). Test specimens 15 mm wide were cut from the lap joints perpendicularly to the welding direction. To offset the axes of the lap specimens, two 6-mm-thick supporting plates were used in the lap shear tests, as shown in Figure 4. The shear fracture strength of each sample was calculated by dividing the fracture load by the sample width (N/mm). Three trials were performed for each configuration. The resulting shear failure strength reported is the average value. After the lap shear tests, the fracture positions were recorded for each lap joint. A scanning electron microscope (JSM-7001F, JEOL, Tokyo, Japan) was used to duplicate the fracture morphology under an accelerating voltage of 15 kV.

## 3. Results

### 3.1. Transverse Sections of the Lap Joints

**Figure 5:**
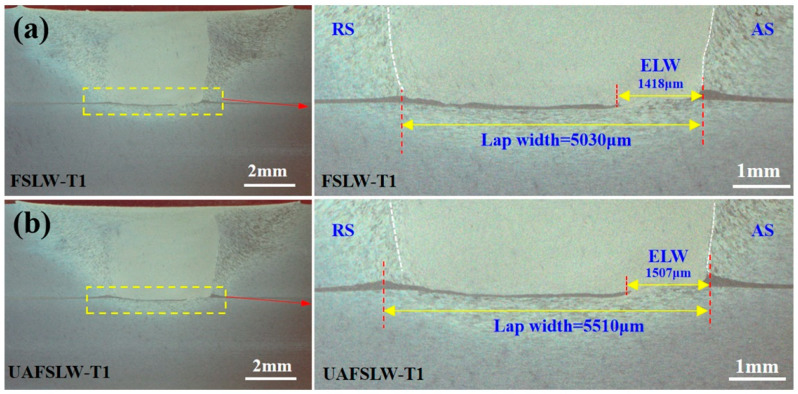

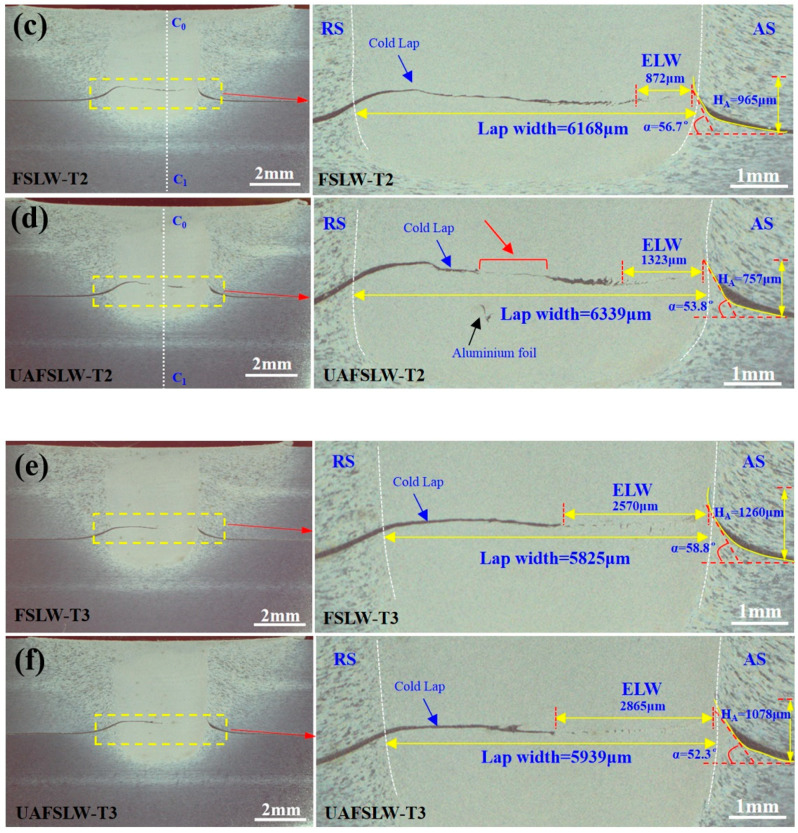
Transverse cross section of lap joints obtained by the different tools (**a**,**b**) friction stir lap welding (FSLW) and ultrasonic-assisted friction stir lap welding (UAFSLW) welded by tool T1, (**c**,**d**) FSLW and UAFSLW welded by tool T2, and (**e**,**f**) FSLW and UAFSLW welded by tool T3.

Figure 5 compares transverse sections of FSLW and UAFSLW joints fabricated with the three different tools. As indicated, the joints are well formed under all conditions with no voids or other common defects. The addition of the aluminium foil as marking material makes the flow characteristics of the metal at the lap joint more apparent. The frictional heat generated between the shoulder and the sheet and the intense stirring of the tool rapidly alters the material of the SZ into a plasticised state. The titling angle and the tool thread cause the metal to migrate in the thickness direction, severely deforming the aluminium foil in the middle layer of the lap joint upon stirring by the tool. Figure 5a,b show that, when the pin length is relatively short (6 mm), the pin fails to completely penetrate the lower sheet. Because the pin length is similar to the thickness of the upper sheet, the material around the bottom of the pin is strongly compressed, leading to a large flow velocity and a concomitant thinning of the aluminium foil at the interface. The interface of the lap joint welded by T1 is magnified and the outline of the SZ is traced with white lines. The ELW generally refers to the horizontal distance between the tips of the hook defect on the AS and the cold lap defect on the RS. In this study, the aluminium foil continuously distributed along the lap interface was regarded as the cold lap defect. The ELW was defined as the horizontal distance between the tips of the hook defect and the continuously distributed aluminium foil, i.e., the width of the vanishing zone of aluminium foil, as marked in Figure 5a,b.

For FSLW and UAFSLW lap joints welded by tools T2 and T3, the pin was completely inserted into the lower sheet. Two geometric defects appeared on the interface of the joint. Hook defects on the AS appeared only in the thermo-mechanically affected zone (TMAZ), and cold lap defects on the RS appeared in the SZ. During welding, the original interface material is stirred and, thus, moves up and down, forming hook defects and cold lap defects. When using tools T2 and T3 for welding, the aluminium foil feature at the interface was significantly thinner and more intermittent than when using tool T1. When tool T2 was used for welding, the aluminium foil in the SZ of the UAFSLW joint was broken, forming a wider vanishing zone of aluminium foil, as shown by the red arrow in Figure 5d. In addition, scattered aluminium foil (shown by the black arrows in Figure 5d) was observed adjacent to the vanishing zone of the aluminium foil, which indicates that ultrasonic vibration shears the aluminium foil at the interface layer, causing the broken foils to flow with the plasticised metal. The migration distance of the hook defect on the AS is defined as H_A_. Previous results show that the defect morphology and H_A_ significantly affect the lap shear-failure load and the failure mode of lap joints, which is attributed to hook defects and cold lap defects concentrating local stress and promoting crack propagation when tense. In general, a large hook defect bending angle (α), a large H_A_, and continuously distributed cold lap defects within the SZ significantly degrade the mechanical properties.

**Figure 6:**
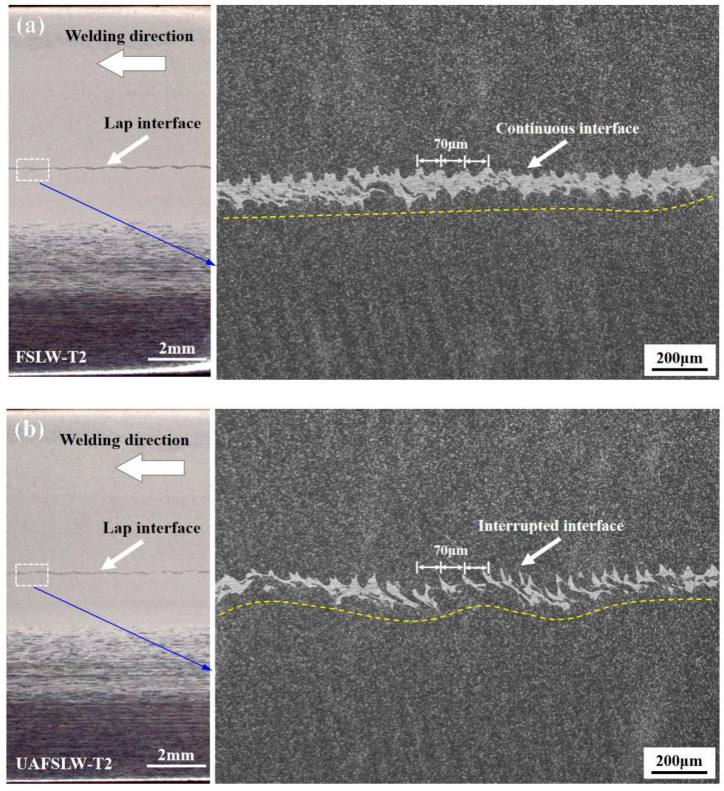
Longitudinal cross section (C_0_–C_1_) of lap joints produced by tool T2: (**a**) FSLW joint and (**b**) UAFSLW joint.

**Figure 7:**
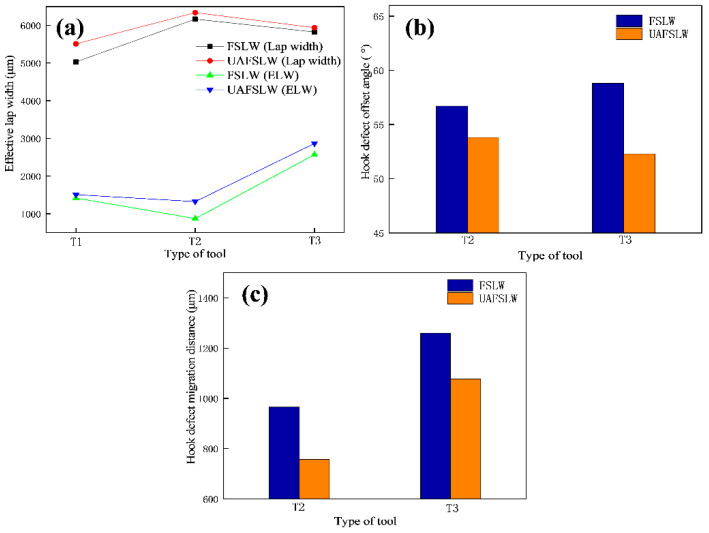
(**a**) Effective lap width (ELW), (**b**) α, and (**c**) H_A_ of the FSLW and UAFSLW joints in Figure 5.

Figure 6 shows the longitudinal cross section of lap joints fabricated by tool T2. Specifically, the joints are observed along the segment C_0_–C_1_ of the centre line of the joints (see Figure 5c,d). These results show clearly that the interface of the FSLW joint is relatively straight and continuous, whereas the interface of the UAFSLW joint is more tortuous and interrupted. The two joints have the same banding spacing, which equals the advancement of the tool for each rotation (70 μm). The ELW and defect size are summarised in Figure 5, as shown in Figure 7. Under the same welding conditions, ultrasonic vibrations tended to increase both the ELW and the lap width, indicating that ultrasonic vibration promotes the flow of interface material and enlarges the width of the bonding between the upper and lower sheets. In addition, the offset angle and migration distance of the hook defect of the UAFSLW joint were slightly smaller than those of the FSLW joint. This may have been caused by the axial ultrasonic vibration reducing the metal deformation resistance of the SZ and the TMAZ.

**Figure 8:**
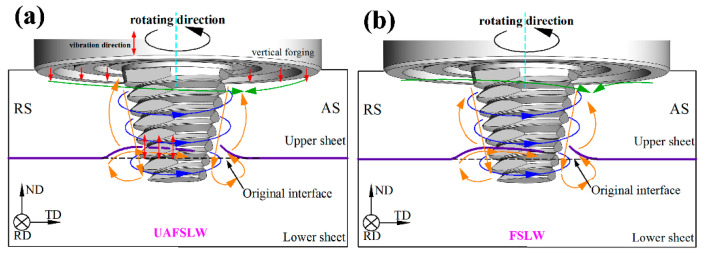
Schematic showing material flow behaviour during UAFSLW and FSLW. (**a**) UAFSLW; (**b**) FSLW.

Based on the transverse sections of the lap joints in Figure 5, Figure 8 shows a schematic of material flow in the FSLW and UAFSLW joints during welding. The flow of plastic material in the vicinity of the tool can be separated into three components: rigid-body rotation, uniform translation, and ring-vortex circulation [30]. As shown in Figure 8, the metal around the pin is subjected to a high shear stress on the surface of the tool, and the rigid-body rotation is indicated by the blue arrows. Given that the material is sheared and forged by the shoulder, the translation of the material fills the cavity in the AS, as indicated by the green arrows. Threads on the pin and the tilt of the tool force metal to migrate in the plate-thickness direction, forming the ring-vortex circulation feature, which is indicated by orange arrows. The various flow-mode patterns in the SZ deform the original interface between the upper and lower sheets (purple lines). Axial ultrasonic vibration of the tool causes high-frequency axial vibration of the shoulder and pin, and the forging action of the shoulder promotes migration of the metal in the thickness direction. Similarly, the lap interface is broken by the pin, and the cold lap defects become intermittently distributed.

### 3.2. Microstructure of Lap Joints

**Figure 9:**
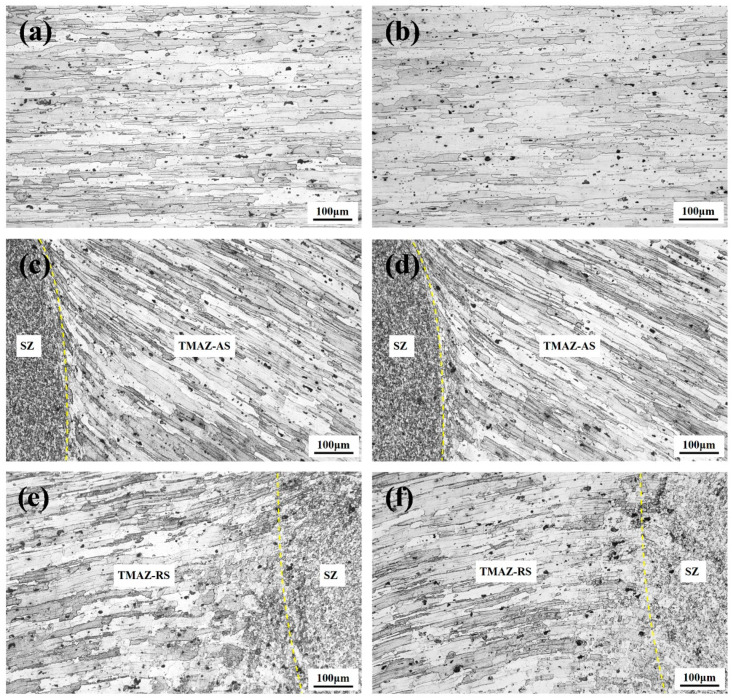
Microstructural images of the (**a**) base material, (**b**) heat-affected zone, (**c**) TMAZ on AS of the FSLW joint, (**d**) TMAZ on AS of the UAFSLW joint, (**e**) TMAZ on RS of the FSLW joint, and (**f**) TMAZ on RS of the UAFSLW joint.

**Figure 10:**
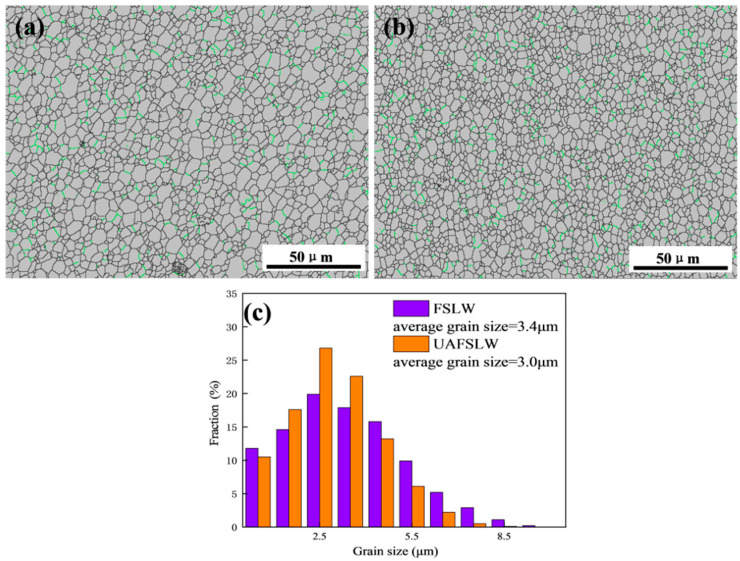
Grain boundary maps in the stirred zone (SZ) of FSLW and UAFSLW joints welded by tool T2: (**a**) FSLW, (**b**) UAFSLW, and (**c**) grain size distribution.

Figure 9 shows the microstructure of the base material, heat-affected zone, and TMAZ of the FLSW and UAFSLW joints welded by tool T2. The base material is characterised by elongated grains produced by rolling, as shown in Figure 9a. Heat cycling during welding makes grains slightly coarser than the base material in the heat-affected zone (Figure 9b). The material in the TMAZ underwent thermal cycle and mechanical stirring, which deformed the grains (Figure 9c,d). The more severe deformation of the AS than the RS causes more severe grain deformation in the TMAZ on the AS than on the RS. The microstructure in the TMAZ of the FSLW joint is similar to that of the UAFSLW joint. Figure 10 shows grain boundary maps of the SZ of the FSLW and UAFSLW joints. The intense plastic deformation and high frictional temperature during welding causes dynamic recrystallisation in the SZ, forming uniform fine-equiaxial grains [31]. The average grain size in the SZ of the FSLW joint is 3.4 μm, which is slightly larger than that for UAFSLW joints (3.0 μm), mainly because of the increased strain rate due to the added ultrasonic energy, which promotes the formation of sub-grains and recrystallisation nucleation and refines the grains [32,33].

### 3.3. Mechanical Properties of Lap Joints

**Figure 11:**
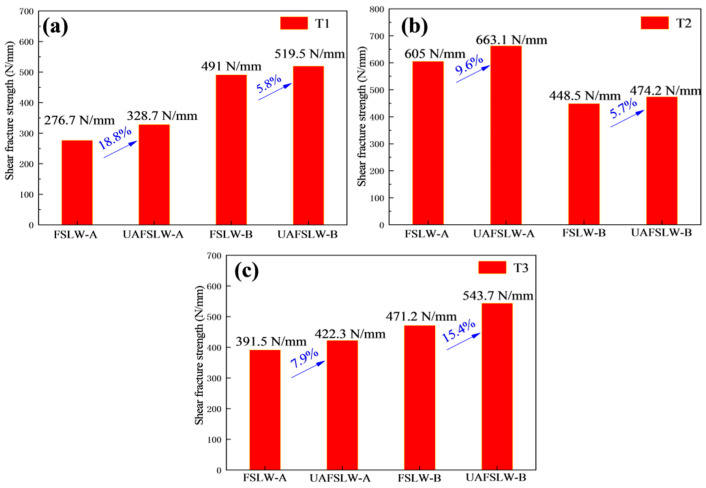
Shear fracture strength of FSLW and UAFSLW joints that were welded using (**a**) tool T1, (**b**) tool T2, and (**c**) tool T3.

Figure 11 shows the shear fracture strength of FSLW and UAFSLW joints fabricated with the three different tools and under different configurations. The shear fracture strength of the UAFSLW joints is greater than that of the FSLW joints in both configurations. In configuration A, the maximum shear fracture strengths for both FSLW and UAFSLW joints are obtained when using tool T2, whereas the minimum fracture strength of the FSLW and UAFSLW joints are obtained when using tool T1. This result may be attributed to the small plunge depth of the pin, which produces in a thinner ELW. In configuration B, the maximum shear fracture strengths for both FSLW and UAFSLW joints are obtained when using tool T3. However, the shear fracture strength of the joint increases upon applying ultrasonic vibration, and the increase in the shear fracture strength of the joints welded by the tool T1 is the largest. This is mainly attributed mainly to the ultrasonic vibration refining the grain size of the SZ and weakening the interface defects (the ELW increases and the hook decreases), increasing the shear fracture strength of the joint.

### 3.4. Fracture Features of the Lap Joints

**Figure 12:**
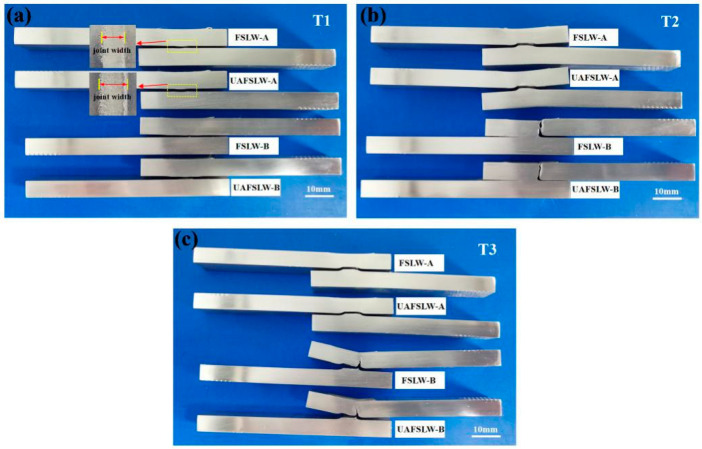
Fracture positions of FSLW and UAFSLW joints: (**a**) FSLW and UAFSLW welded by tool T1, (**b**) FSLW and UAFSLW welded by tool T2, and (**c**) FSLW and UAFSLW welded by tool T3.

Figure 12 shows the fracture positions in the FSLW and UAFSLW joints after lap shear tests. In addition to the joints welded by tool T1, the fracture positions in the FSLW and UAFSLW joints fabricated with the tools T2 and T3 differ greatly for both configurations A and B. This result is mainly attributed to the pin length of tool T1 being almost the same as the top-sheet thickness, so that the joint is not sufficiently wide, resulting in a fracture at the interface between the upper and lower sheets. For the joints welded by tools T2 and T3, even though the fracture positions within the joints differ for both configurations A and B, the fracture positions within the FSLW and UAFSLW joints are the same for the same configuration conditions, indicating that the fracture position of the joint is not affected by ultrasonic vibration.

**Figure 13:**
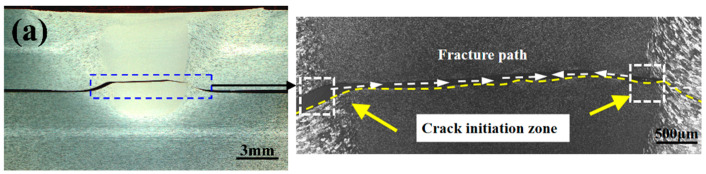

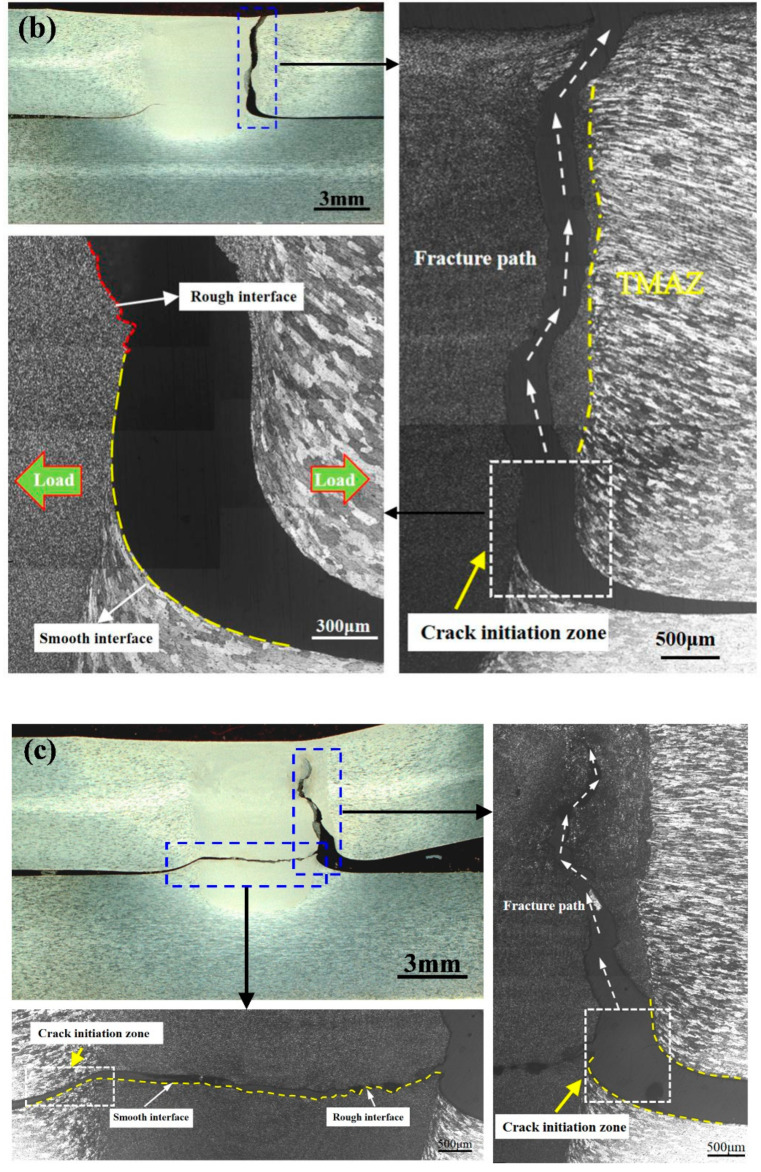
Fracture modes of FSLW joints: (**a**) fracture mode I, (**b**) fracture mode II, and (**c**) fracture mode III.

Figure 13 shows the characteristic fracture behaviour of the FSLW joints, and three fracture modes occur during lap shear tests. Fracture mode I is a shear fracture where the failure occurs along the original interface of the whole joint. For both configurations A and B, only shear-fracture mode occurs for the FSLW and UAFSLW joints when using tool T1. Shear fracture also occurs for the FSLW and UAFSLW joints fabricated with tools T2 and T3 in configuration A (Figure 13a). Fracture mode II is a tensile fracture where the crack initiates at the tip of the hook defect on the AS, and the end of the fracture path is nearly normal to the tensile direction. This fracture mode occurs in the FSLW and UAFSLW joints fabricated by tool T2 in configuration B (Figure 13b). The fracture path propagates up the SZ or TMAZ interface until tensile fracture occurs. Although the metal around the hook defect is severely deformed, the interface hook hinders the mixing of the metals on both sides, so the interface remains very smooth after the fracture, and the rough interface of the SZ and TMAZ exhibits typical tensile-fracture characteristics. Fracture mode III is a mix of shear fracture and tensile-fracture modes (Figure 13c), where the joint fracture occurs at the lap interface of the upper and lower sheets. However, two crack sources are active. One is the hook defect of the AS, where the crack propagates upward along the tip of the hook, and the other is the cold lap defect of the RS, where the crack propagates horizontally along the lap interface. Fracture mode III mainly occurs in FSLW and UAFSLW joints fabricated in configuration B by tool T3.

**Figure 14:**
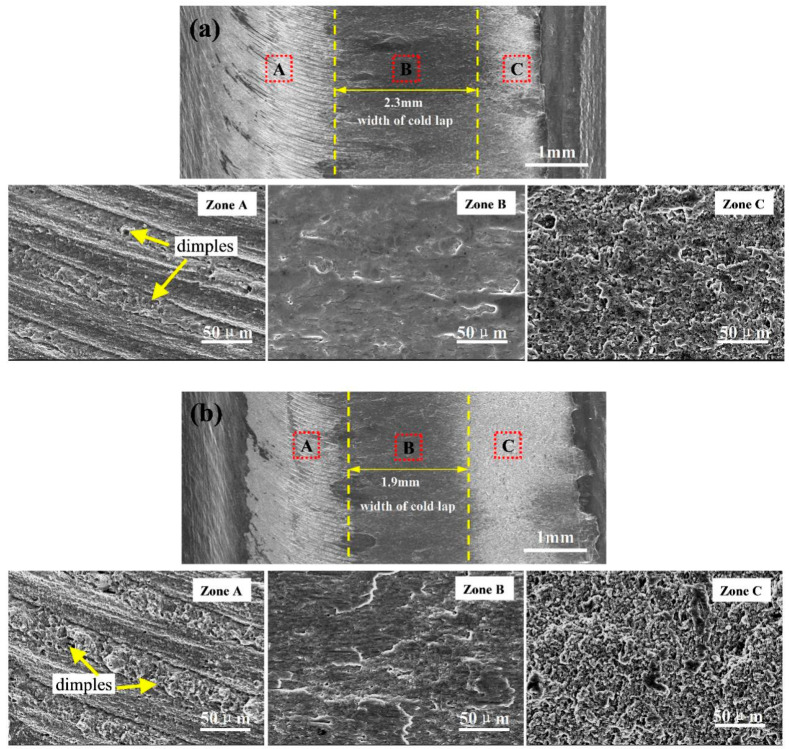
Fracture morphologies of the FSLW and UAFSLW joints welded by tool T2 under the lap configuration A: (**a**) FSLW and (**b**) UAFSLW.

**Figure 15:**
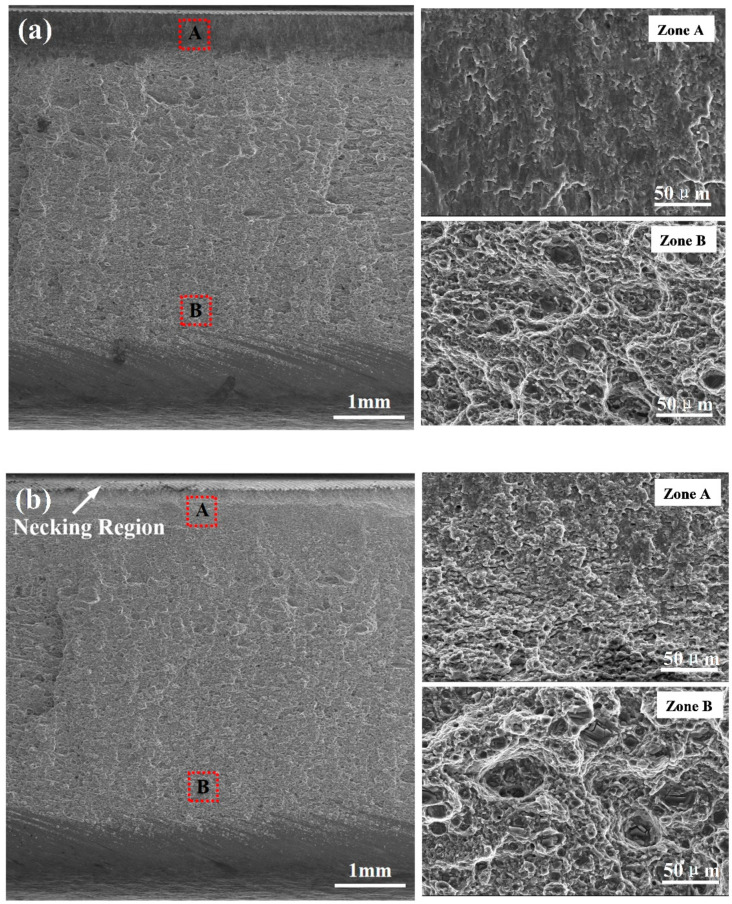
Fracture morphologies of the FSLW and UAFSLW joints welded by tool T2 in lap configuration B: (**a**) FSLW and (**b**) UAFSLW.

To improve the mechanical properties of lap joints by using axial ultrasonic vibration, we study the fracture morphologies of the FSLW and UAFSLW joints fabricated using tool T2 (see Figure 14 and Figure 15). Figure 14 shows the shear-fracture morphologies of the FSLW and UAFSLW joints in configuration A. Figure 14a,b show a general view of the lower sheet of the FSLW and UAFSLW joints, respectively. The fracture morphologies of the FSLW and UAFSLW joints can be divided into three zones. Zone A contains arc-shaped stripes with some dimples on the AS. The dimples form due to the movement of the metal of the upper and lower sheets in the vertical direction, indicating good diffusion bonding. Compared with FSLW joints, the ultrasonic vibration and concomitant mixing of the material produces more dimples (zone A). No clear dimples appear at the centre of the zone, even though some flat areas appear in zone B with partial brittle fracture characteristics. However, ultrasonic vibration significantly reduces the width of the cold lap defect, thereby improving the mechanical properties of the joint. Pits of different depths produce a mixed fracture mode of toughness and brittleness in zone C, indicating that cold lap defects weaken the bonding strength of the upper and lower sheets.

Figure 15 shows the tensile-fracture morphologies of the FSLW and UAFSLW joints welded by tool T2 in configuration B. Compared with the FSLW joint (Figure 15a), a necking region appears in the upper part of the fracture of the UAFSLW joint (Figure 15b), indicating that ultrasonic vibration improves the toughness of the lap joint. The fracture in the FSLW and UAFSLW joints can be divided into two parts. The magnified images of zone A show the brittle fracture feature of the flat in the FSLW joint, whereas the ductile fracture mode of pits of various sizes and depths occurs in the UAFSLW joint. The lower part (zone B) in the FSLW and UAFSLW joints is composed of numerous dimples containing fragmented second-phase particles. The reduced metal flow resistance due to ultrasonic vibration leads to sufficient mixing of the metal in the SZ, so that larger dimples and thicker tearing edges appear on the fracture surface of the UAFSLW joint.

## 4. Discussion

**Figure 16:**
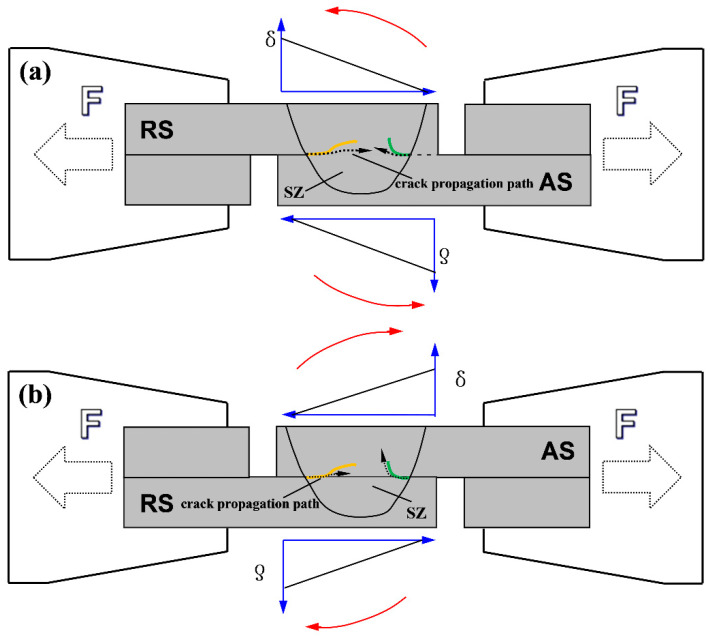
Schematic views of tensile fracture behaviour of lap joints in configuration (**a**) A and (**b**) B.

The results indicate that the shear fracture strength of lap joints is mainly related to the interface defects and the size of the ELW. In addition to the tools used, the different configurations affect the fracture mode. Schematic views of the tensile fracture of lap joints for both configurations are shown in Figure 16, where hook and cold lap defects are shown by green and yellow lines, respectively, and the tensile stress and crack-propagation paths during tensile testing are indicated by black solid and dashed arrows, respectively. Based on Figure 12, the joints welded in configuration A were more susceptible to fracture mode I (i.e., shear fracture), whereas the joints mainly underwent fracture mode II (tensile fracture) and fracture mode III (mixed fracture) in configuration B.

In configuration A, the shear fracture strength of the joint was proportional to the ELW. The ELW of the joint obtained by tool T2 was the greatest, as it extended the propagation path of the crack and its shear fracture strength was also the highest. In configuration B, the shear fracture strength of the joints varied with each of the three tools and was mainly related to the fracture behaviour of the joints. The hook defect of the joint obtained by the T2 tool was perpendicular to the direction of the tensile force. Since the materials at the hook defect were not mixed, it was similar to prefabricating a crack defect at the hook defect. During the tensile process, it rapidly expanded upward along the hook defect, worsening the shear fracture strength of the joint. Although the joint obtained by the T3 tool demonstrated a tensile fracture, the joint eventually fractured along the lap interface. These two fracture paths lead to the maximum shear fracture strength. In summary, the concentration of local stress caused by the hook defect deteriorated the mechanical properties of the joint. Ultrasonic vibration was demonstrated to increase the shear fracture strength of the joint due to three main reasons. First, ultrasonic vibration reduces the flow resistance of the SZ material, leading to a greater ELW. Second, the high-frequency vibration pin shears the interface along the axial direction to promote the mixing of the upper and lower sheet metals. Finally, ultrasonic vibration refines the grain size, resulting in a more circuitous crack-propagation path.

## 5. Conclusions

We investigate in this study the joint formation, microstructural characteristics, and mechanical properties of FSLW and UAFSLW joints of the 7075-T6 alloy. The conclusions of significance are drawn as follows.

(1) Axial ultrasonic vibration of the tool expands the size of the SZ and refines the grain size. Ultrasonic vibration breaks up cold lap defects, making them more curved than in traditional FSLW joints and intermittently distributed. Ultrasonic vibration also reduces the flow resistance of the material and the tilt angle and height of the hook defects.

(2) The lap shear test reveals three fracture modes of the joint: shear fracture, tensile fracture, and a mixture of both. With insufficient plunge depth of the tool T1, shear fracture occurs under both configurations A and B. In the A (B) configuration, the fracture mode of the joint is mainly shear (tensile) fracture. For B-configuration joints made by tool T3, the mixed fracture mode is predominant.

(3) Application of axial ultrasonic vibration does not change the fracture mode. The lap shear fracture strength of UAFSLW joints exceeds that of traditional FSLW joints by about 5–19% under the same conditions, which is mainly attributed to the fact that axial ultrasonic vibration reduces the size of cold lap defects and the offset angle of hook defects. In configuration A, the maximum lap shear fracture strength is obtained with tool T2, and the maximum shear fracture strength of the UAFSLW joints tested was 663.1 N/mm.

## Figures and Tables

**Table 1:** Chemical composition and mechanical properties of 7075-T6 aluminium alloy.

Chemical Composition (wt %)						Mechanical Properties		
Zn	Mg	Cu	Mn	Fe	Cr	Al	Tensile Strength	Elongation
5.24	2.23	1.41	0.09	0.21	0.24	Bal.	572 MPa	14%
